# Hemoglobin synthesis rates in young trained and untrained males and females are not different

**DOI:** 10.14814/phy2.70311

**Published:** 2025-04-19

**Authors:** Hilkka Kontro, Chris McGlory, Martin J. MacInnis

**Affiliations:** ^1^ Faculty of Kinesiology, Human Performance Lab University of Calgary Calgary Alberta Canada; ^2^ School of Kinesiology and Health Studies Queen's University Kingston Ontario Canada

**Keywords:** absolute synthetic rate, deuterium oxide, exercise physiology, fractional synthetic rate, heavy water, hemoglobin mass, oral stable isotope tracer

## Abstract

We employed deuterated water (^2^H_2_O) to compare the fractional synthetic rate (FSR) of hemoglobin in trained (*n* = 10) and untrained humans (*n* = 10). Participants had a mean V̇O_2_max of 49.8 [SD: 10.9] mL/kg/min, hemoglobin mass of 775 [180] g, and red blood cell volume of 2370 [550] mL. After a loading dose, participants ingested ^2^H_2_O daily for 28 days to maintain a stable ^2^H body water enrichment (~0.5 atom percent excess (APE)), as measured in saliva samples. ^2^H‐enriched alanine APE was measured in RBC protein using gas chromatography pyrolysis isotope ratio mass spectrometry. The increase in APE for Hb protein was nonlinear for the first 2 weeks but stabilized from day 14 to day 28, with mean APE reaching 0.033 [0.005]% on day 28. Hb FSR calculated over this 2‐week period was 0.84 [0.15]%/day, which equated to an Hb absolute synthetic rate of 6.5 [2.2] g/day and a lifespan of 126 [30] days. Hb FSR was not different between trained (0.83 [0.19] %/day) and untrained (0.86 [0.24] %/day, *p* = 0.81) individuals or between males (0.80 [0.25] %/day) and females (0.88 [0.17] %/day, *p* = 0.24). Endurance training and sex did not affect Hb FSR. The ^2^H_2_O method to measure Hb FSR is feasible in humans.

## 1 INTRODUCTION

Chronic endurance training is associated with elevations in blood volume and hemoglobin mass (Hb_mass_) (Heinicke et al., 2001; Kjellberg et al., 1949; Montero et al., 2017; Schmidt & Prommer, 2008) that contribute to enhanced maximal oxygen uptake (V̇O_2_max) (Hagberg et al., 1998; Kanstrup & Ekblom, 1982). Red blood cells (RBCs) are synthesized in bone marrow, with an expected lifespan of approximately 120 days; however, the RBC lifespan for endurance athletes is reported to be shorter than that of untrained individuals (Mairbäurl et al., 1983; Weight et al., 1991), which would necessitate a faster RBC turnover rate. Hyperplasia of the hematopoietic bone marrow has also been reported in endurance athletes (Caldemeyer et al., 1996; Shellock et al., 1992; Vogt et al., 2008), which may facilitate a higher rate of erythropoiesis than in untrained humans. The methods available to study RBC production in humans are complex and protracted, limiting insights into the regulation of erythropoiesis.

The deuterated water (^2^H_2_O) oral stable isotope method (Gasier et al., 2010) can be used to measure hemoglobin (Hb) fractional synthetic rates (FSR) in humans (Kontro et al., 2025). When ^2^H_2_O is consumed, hydrogens on amino acids are labeled with the deuterium isotope through in vivo transamination reactions (Miller et al., 2020). Subsequently, the rate of incorporation of the labeled amino acids into the protein of interest (i.e., Hb) over time is used to calculate FSR (Miller et al., 2020; Wilkinson et al., 2017). The technique involves sampling saliva (a surrogate for the precursor pool; (Lukaski & Johnson, 1985)) and hemoglobin (which comprises 97% of RBC protein (Weed et al., 1963)) at specific time points. For humans, almost all of the proteins in RBCs are synthesized during maturation in the bone marrow (Palis, 2014), meaning there is a delay between the synthesis of Hb and its appearance in circulation that necessitates a relatively prolonged sampling period (e.g., weeks). Still, the duration of this protocol is considerably shorter than most other RBC cohort labeling methods (e.g., ^15^N, biotin), which typically require several months of blood sampling, reducing the burden for participants and the impact of potential tracer loss and recycling (Wagenmakers, 1999). Furthermore, because Hb_mass_ (i.e., the total protein pool) can be measured in humans (Burge & Skinner, 1995), it is also theoretically possible to measure the Hb absolute synthetic rate (ASR) using this method. Given its advantages, this ^2^H_2_O technique can be applied to investigate many areas of human physiology, including the influence of aerobic fitness on Hb synthesis rates.

The aim of the present study was to determine whether there were differences in Hb FSR and ASR between habitually trained and untrained individuals. We hypothesized that Hb FSR and ASR would be higher in trained humans in line with a reduced RBC lifespan and greater Hb_mass_. We also sought to explore potential sex‐related differences in blood protein synthesis rates based on differences in Hb_mass_ (Goodrich et al., 2020; Kontro et al., 2024) and iron status (Finch & Cook, 1984) between males and females.

## 2 METHODS

### 2.1 Ethical approval

All participants provided written, informed consent prior to enrollment in the study and were required to pass the Physical Activity Readiness Questionnaire (PAR‐Q+). The study was approved by the University of Calgary Conjoint Health Research Ethics Board (REB19‐0215) and conformed to the standards set by the Declaration of Helsinki, except for registration in a database.

### 2.2 Study design

This cross‐sectional study investigated Hb synthesis in healthy endurance‐trained and untrained males and females during 4 weeks of habitual living. Participants completed an aerobic fitness test and a Hb_mass_ assessment prior to baseline sample collection. Thereafter, participants consumed ^2^H_2_O daily for 4 weeks to assess Hb FSR and ASR (Kontro et al., 2025). Regular blood and saliva samples were collected during this period, and Hb_mass_ was assessed again at the end of the study. Participants were required to maintain their typical training habits throughout the study and keep a log of all their training activities. Albumin FSR was also assessed as an internal control.

The RBC lifespan was used as a proxy for Hb turnover for statistical power calculations. Based on the results of Weight et al. (1991), where trained runners and untrained controls had RBC lifespans of ~70 [25] days and 114 [30] days, we calculated an effect size of 1.6. The required sample size (*α* = 0.05 and 1 – *β* = 0.80) for this effect size was eight participants per group (16 in total), and 20 participants were recruited to account for participant attrition.

### 2.3 Participants

Twenty volunteers (10 F, 10 M) partook in the study, with equal numbers of trained and untrained participants within each sex. Females and males were approximately matched for aerobic fitness (Tripp et al. 2025) when V̇O_2_ max was normalized to fat‐free mass (Table 1; *p* = 0.57). Over the preceding 3 months, self‐reported habitual endurance training volume was 8.4 [2.9] h/week for the trained group and 1.2 [1.0] h/week for the untrained group. The trained participants had been involved in endurance sports for 10 [6] years. Participants' primary sports were cycling (*n* = 4), running (*n* = 2), triathlon (*n* = 2), cross‐country skiing (*n* = 1), and rowing (*n* = 1). For the untrained group, an activity level corresponding to the minimum suggestion of the ASCM physical activity guidelines (~150 min/week of moderate‐vigorous exercise) or less was accepted with no history of competitive endurance sports. Both eumenorrheic females (*n* = 4) and females on hormonal contraceptives (*n* = 6) were included. No participants were amenorrheic.

**TABLE 1:** Participant characteristics at baseline disaggregated by sex and training status.

	All (*n* = 20)	Trained (*n* = 10)	Untrained (*n* = 10)	Males (*n* = 10)	Females (*n* = 10)	*p* Value (training status × sex; training status; sex)
Age (y)	28 [6]	30 [6]	26 [7]	29 [8]	27 [4]	0.78; 0.28; 0.34
BM (kg)	66.2 [10.9]	65.8 [11.4]	66.7 [11.0]	73.7 [10.2]*	58.8 [3.1]	0.39; 0.80; **0.001**
FFM (kg)	51.3 [10.8]	52.5 [11.3]	50.1 [10.8]	59.1 [10.1]*	43.5 [3.1]	0.92; 0.50; **<0.001**
Hb_mass_ (g)	775 [180]	815 [193]	753 [166]	902 [157]*	648 [89]	0.96; 0.18; **<0.001**
Hb_mass_ (g∙kg^−1^)	11.6 [1.4]	12.3 [1.4]*	10.9 [1.0]	12.2 [1.5]*	11.0 [1.0]	0.33; **0.008**; **0.013**
Hb_mass_ (g∙kg FFM^−1^)	15.1 [1.7]	15.6 [2.1]	14.7 [1.2]	15.4 [1.9]	14.9 [1.5]	0.94; 0.27; 0.54
[Hb] (g∙dL^−1^)	15.2 [1.3]	15.2 [1.3]	15.5 [1.5]	16.2 [1.0]*	14.2 [0.6]	0.45; 0.09; **<0.001**
V̇O_2_max (mL∙kg^−1^∙min^−1^)	49.8 [10.9]	58.8 [6.0]*	40.7 [5.4]	53.1 [11.2]*	46.4 [10.0]	0.47; **<0.001**; **0.006**
V̇O_2_max (mL∙kg FFM^−1^∙min^−1^)	64.3 [12.5]	74.1 [8.3]*	54.4 [6.9]	65.9 [11.5]	62.6 [13.9]	0.68; **<0.001**; 0.36
Training volume (h ∙wk^−1^)	4.9 [4.4]	8.6 [3.0]*	1.2 [1.0]	4.8 [4.4]	5.0 [4.2]	0.34; **<0.001**; 0.67

*Note*: Data are reported as mean [SD]. Bold: *p* < 0.05. The asterisk (*) indicates the significantly higher mean within a factor.

Abbreviations: BM, body mass; FFM, fat‐free mass; Hb, hemoglobin; Hb_mass_, hemoglobin mass; V̇O_2_max, maximal oxygen uptake.

### 2.4 Data collection

#### 2.4.1 Training load

Participants wore a heart rate (HR) monitor (4iii, Cochrane, AB, Canada) and provided their session Rating of Perceived Exertion values (sRPE) for all exercise training sessions. The 4‐week training load of the participants was assessed using total training volume (hours) and sRPE values (Foster et al., 2001).

#### 2.4.2 Body composition

Whole‐body dual‐energy X‐ray absorptiometry (DXA) scans (Lunar iDXA, GE Healthcare, Chicago, IL, USA) were used to quantify fat‐free mass (FFM).

#### 2.4.3 Ramp incremental tests

To assess aerobic fitness, a ramp incremental test was conducted on an electromagnetically braked cycle ergometer (Velotron; Dynafit Pro, Racer Mate, Seattle, WA, USA). After a 4‐min warm‐up at 50 W, a ramp of 30 W/min (for trained) or 20 W/min (for untrained) began, and the participants pedaled until volitional exhaustion. Ventilatory and gas exchange variables were measured with a metabolic cart connected to a mixing chamber (Quark CPET, Cosmed, Rome, Italy) with 10‐s data averaging. The flowmeter and gas analyzers were calibrated prior to each test following the manufacturer's instructions. HR was monitored and recorded continuously using a chest strap HR monitor (Polar Electro, Kempele, Finland). V̇O_2_max was defined as the highest 30‐s rolling average of V̇O_2_.

#### 2.4.4 Hb_mass_ and vascular volumes

Hb_mass_ was measured before and after the 4‐week protocol using a modified CO rebreathing procedure, as described by Schmidt & Prommer (2005). Briefly, 0.8–1.2 mL/kg body mass (BM) of CO (determined based on sex and habitual physical activity) was injected into a closed respirometer system (Blood tec GmbH, Bayreuth, Germany) connected to a 3‐L anesthetic bag filled with 100% O_2_, and this gas mixture was rebreathed for 2 min. Before the rebreathing and 7 min after the start of rebreathing, blood samples were drawn from a forearm vein. Carboxyhemoglobin concentration ([COHb]) and [Hb] were measured using a blood gas analyzer (ABL80 FLEX; Radiometer, Brea, CA). CO from exhaled air and remaining in the respirometer was measured using a hand‐held CO detector (Dräeger Pac 7000, Dräeger AG, Lübeck, Germany) connected to a mouthpiece. Blood volume (BV), plasma volume (PV), and red blood cell volume (RBCV) were calculated as described previously by our lab (Kontro et al., 2022).

### 2.5 ^2^H_2_O protocol

#### 2.5.1 Dosing and blood sampling overview

^2^H_2_O (Cambridge Isotope Laboratories, Inc., MA, USA; CAS# 7789‐20‐0) was administered based on FFM at a dose of 0.625 mL/kg FFM for a single serving (Oikawa et al., 2020). On Day 0, baseline blood samples and saliva were collected. Thereafter, a loading dose of 8 servings (only on day 0) of ^2^H_2_O was provided for the participants to ingest at 90 min intervals during the day. Participants consumed one dose per day for the next 28 days.

On Days 1, 3, 7, 14, 21, and 28, the participants returned to the laboratory for fasted morning blood samples. On either day 1 or day 3, additional blood samples were obtained with appropriate tubes to measure markers of iron status and a complete blood count using a clinical laboratory.

#### 2.5.2 Sample processing

Blood was drawn into a 2.7‐mL Na^+^‐citrate plastic blood collection tube (BD Vacutainer, BD, Franklin Lakes, NJ, USA) using standard procedures and centrifuged at 1500*g* for 10 min at +4°C. Plasma was aliquoted into tubes and frozen at −30°C until sample preparation.

#### 2.5.3 Hemoglobin isolation

After the initial centrifugation, 400 μL from the RBC fraction was pipetted into a 1.5 mL tube and gently washed with an equal volume of cold PBS. The solution was centrifuged at 10000*g* for 5 min at 4°C, and the supernatant was discarded. The remaining cells were stirred with the pipette tip and aspirated slowly several times until the suspension appeared homogeneous. After repeating this procedure three times, the washed RBCs were lysed with two cycles of freezing (15 min, −80°C, followed by thawing on ice), and then homogenized by sonication at +4°C (20 min, 17 W). The lysed cells were centrifuged at 10000*g* for 15 min at +4°C, and the Hb‐containing supernatant was collected and used for HCl hydrolysis.

#### 2.5.4 Albumin isolation

**FIGURE 1:**
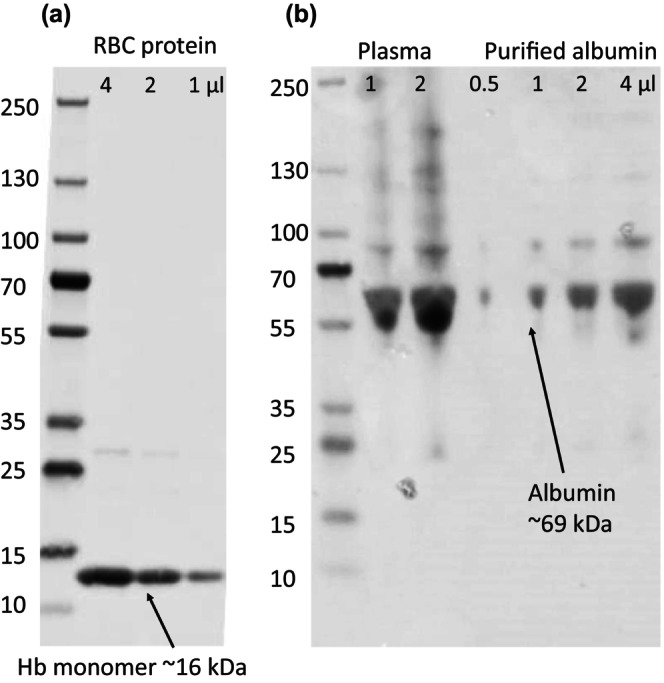
A representative image of gel electrophoresis of (a) washed and hydrolysed red blood cells, and (b) plasma versus purified albumin from one participant. The proteins from a 4% to 15% TGX stain‐free gel were transferred to a nitrocellulose membrane and stained with Ponceau S for imaging.

Plasma proteins were precipitated from 500 μL plasma by adding 1 mL 10% trichloroacetic acid (TCA) (wt:vol) and then centrifuging at 4500*g* for 5 min. The supernatant was discarded, and the resultant pellet was resuspended in 500 μL ddH_2_O. Albumin was solubilized by adding 2.5 mL 1% TCA (wt:vol) in 90% ethanol and centrifuged at 4500*g* for 5 min. The supernatant fluid was collected, and the albumin was precipitated by adding 1 mL 26.8% ammonium sulfate (wt:vol). After centrifugation at 4500*g* for 5 min, the supernatant was removed and discarded, and the resultant pellet was washed once more with 1 mL 26.8% ammonium sulfate. To remove any residual free amino acids, the albumin pellet was washed twice with 1 mL 0.2 M perchloric acid. The pellet was used directly for HCl hydrolysis. The purity of both Hb and albumin fractions was confirmed by SDS‐PAGE followed by transfer to a nitrocellulose membrane and Ponceau S staining (Figure 1).

#### 2.5.5 HCl hydrolysis and sample purification

The protein‐bound amino acids in both albumin and Hb samples were liberated by HCl hydrolysis. Briefly, 2 mL of Dowex in 1 M HCl (constantly under magnetic stirring) was added to the samples, which were then vortexed and heated in an oven at 110°C for 72 h and vortexed every 24 h. Ion exchange was performed by binding the samples to columns of Dowex, preconditioned with 2 M NH_4_OH and 1 M HCl, then washed with H_2_O until neutral and eluted with 2 M NH_4_OH into glass tubes. The eluted samples were dried in a SpeedVac SPD1030 (Thermo Scientific, USA) at 60°C for 18–24 h, and the resultant amino acid residue was reconstituted in 0.5 M HCl. The final samples were derivatized to their N‐methoxycarbonyl methyl esters and analyzed using gas chromatography pyrolysis isotope ratio mass spectrometry by Metabolic Solutions Inc. (Nashua, NH, USA). Standard curves were obtained separately for each batch of samples (each participant) by injecting three concentrations of in‐house standard blends twice. The resulting slope and intercept were used to derive the corrected value for deuterium, used to calculate atom percent (Equation 3).

#### 2.5.6 Measurement of ^2^H in body water

Saliva samples were taken independently by the participants using Salivette cotton swab tubes (Sarstedt, Nümbrecht, Germany) and frozen immediately. The frozen samples were thawed at room temperature and centrifuged at 1500*g* for 10 min at 4°C to collect the fluid, which was frozen until analysis. The samples were prepared for ^2^H enrichment analysis by diluting them 1:35 in distilled water and vortexing. ^2^H enrichment of saliva was determined by cavity ring‐down spectroscopy using a liquid isotope analyzer (Picarro L2130‐I analyser, Picarro, Santa Clara, CA) with an automated injection system. Each saliva sample was injected six times, with the average of the final three values taken as the final value. The coefficient of variation from the last three samples was <3%.

### 2.6 Data analysis

#### 2.6.1 Calculation of FSR and ASR

**FIGURE 2:**
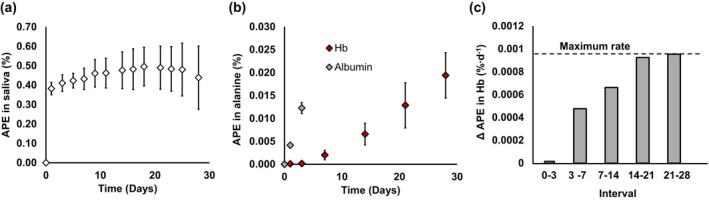
Determination of the linear phase of atom percent excess (APE) increase in Hb. (a) APE in body water (+ SD) over the study period. (b) APE in alanine (%) by sampling days in albumin and hemoglobin (Hb). (c) The rate of change in APE between sampling days for Hb. The maximum rate, measured between days 21 and 28 is shown as a dotted horizontal line. *n* = 20 for all panels.

FSR is defined as the rate at which ^2^H‐labeled alanine is incorporated into the protein when normalized to the total abundance of the precursor pool per unit of time. Atom percent excess (APE) of Hb at timepoints of blood sampling was plotted to identify the linear phase of appearance of deuterated Hb, which began on day 14 (Figure 2a,b); therefore, the APE at 14 and 28 days was used for Hb FSR determination. The mean APE for body water of the first 14 days was used for the calculation of Hb FSR to account for delays between synthesis and appearance. Subsequently, FSR and ASR were calculated using the following equations:

(1) $$\mathrm{FSR}\;\left(\%/\mathrm{day}\right)=\left[\left({\text{ΔAPE}}_{\mathrm{Ala}}\right)\right]/\left[\left({\mathrm{APE}}_{\mathrm{BW}}\right)\times t\right]\times 100$$

(2) $$\mathrm{ASR}=\mathrm{FSR}\times \text{Protein pool}\;\left(g\right)$$

where ΔAPE_Ala_ is the difference in deuterium enrichment of protein‐bound alanine, APE_BW_ is the mean precursor enrichment over time, and *t* is the time between blood samples. A ^2^H exchange ratio of 3.7 between body water and free alanine was assumed (Wilkinson et al., 2014). Since the derivative added 11 unlabelled hydrogens, the APE_Ala_ values were corrected by multiplying by a factor of 3.7/(11 + 3.7) = 0.2517.

Atom percent values were calculated as

(3) $$\text{Atom percent}=\frac{100\times \mathrm{AR}\;\left(\delta^{2}H\times 0.001+1\right)}{1+\mathrm{AR}\;\left(\delta^{2}H\times 0.001+1\right)}$$

Where AR is the absolute ratio constant for deuterium (0.00015595).

To obtain the number of days needed for a complete turnover of the body's Hb stores, RBC lifespan was calculated as

(4) $$\mathrm{RBC}\;\text{lifespan}=\frac{100\%}{\mathrm{FSR}\;(\%\cdot d^{‐1})}\;$$

This equation assumes that Hb lifespan is a proxy for RBC lifespan and that the synthesis rate is static.

Albumin FSR was determined from days 1 and 3 only due to the faster turnover of this protein, using the average ^2^H enrichment in body water of the same days, using Equation 1.

#### 2.6.2 Statistical analysis

Data were compared using a 2‐way ANOVA with training status and sex as factors. Significance was accepted at *α* = 0.05. Normality was assessed using the Shapiro–Wilk test. Statistical analyses were conducted in SPSS (version 26; IBM, Armonk, NY). Figures were created using GraphPad (version 10.2; Prism, La Jolla, CA).

## 3 RESULTS

### 3.1 Participant characteristics

Total volume of endurance training during the study period, 8.8 [3.0] h/week in trained and 1.5 [1.2] h/week in untrained participants, was not different from self‐reported baseline (*p* = 0.78 and *p* = 0.40, respectively; Table 1). Relative hematological values separated by training status and sex are shown in Table 1. The trained group had a greater relative Hb_mass_, relative V̇O_2_max (normalized to BM and FFM), and training volume than the untrained group, but there were no differences in age, BM, FFM, absolute Hb_mass_, Hb_mass_ normalized to FFM, and [Hb] (Table 1). On a group level, no change was observed in Hb_mass_ (775 [180] g vs. 781 [202] g) or BV (5.58 [1.04] L vs. 5.53 [1.21] L) over 28 days.

### 3.2 Isotope enrichment and protein synthesis

Saliva APE over the 28‐day study is presented in Figure 2a. The ∆APE of Hb‐derived alanine reached its maximum between days 21 and 28; however, the ∆APE between days 14 and 21 was 97% of this maximum, which was statistically not different from the maximum, indicating a plateau (∆APE for days 14–21: 0.063 [0.035] % and ∆APE for days 21–28: 0.065 [0.021] %, *p* = 0.80) (Figure 2b,c).

**FIGURE 3:**
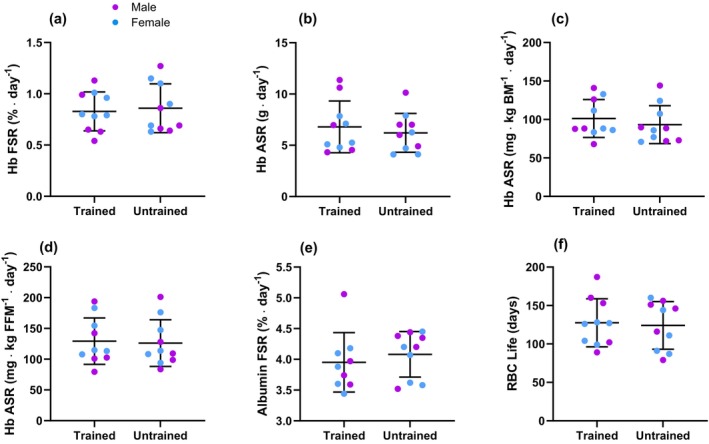
Blood protein synthetic rates for trained (*n* = 10) and untrained (*n* = 10) individuals. Males are represented by purple markers and females by blue markers. (a) Hemoglobin (Hb) fractional synthetic rate (FSR); (b) Hb absolute synthetic rate (ASR); (c) Hb FSR relative to body mass; (d) Hb FSR relative to fat‐free mass (FFM); (e) Albumin FSR; (f) Red blood cell (RBC) lifespan calculated as an inverse of Hb FSR. The middle line represents the mean, and error bars represent one standard deviation. Individual data are shown as circles. *n* = 20 for all panels.

Values for Hb and albumin FSR are reported in Figure 3. Using the linear phase (days 14–28), the mean Hb FSR was 0.84 [21] %/day, with a range of values between 0.54 %/day and 1.27 %/day. These values translated to a mean lifespan of 126 [30] days, with a range of 79–187 days (Figure 3), and an Hb ASR of 6.5 [2.2] g/day. Given that Hb_mass_ was stable in our participants, the synthesis and breakdown rates for Hb were approximately equal: Hb fractional and absolute breakdown rates were 0.82 [0.37] %/day and 6.3 [3.3] g/day for the group. The mean albumin FSR was 4.02 [0.42] %/day.

### 3.3 Effect of training status on Hb FSR

As shown in Figure 3, no differences were observed between trained and untrained participants for Hb FSR (trained: 0.83 [0.19]%/day; untrained: 0.86 [0.24]%/day; *p* = 0.74) and ASR (trained: 6.8 [2.5] g/day; untrained: 6.2 [1.9] g/day; *p* = 0.57), not even when ASR was normalized to body size and expressed as mg/kg BM/day (trained: 101 [25]; untrained: 93 [25]; *p* = 0.48) or as mg/kg FFM/day (trained: 129 [38]; untrained: 126 [38]; *p* = 0.85). Albumin FSR was also not different in trained (4.0 [1.3]%/day) and untrained (4.1 [0.4]%/day) participants (*p* = 0.51).

### 3.4 Effect of sex on Hb FSR

Compared to females, males had a greater BM, FFM, Hb_mass_ (absolute and relative to BM), [Hb], V̇O_2_max (relative to BM), but there were no differences between sexes for age, Hb_mass_ (relative to FFM), V̇O_2_max (relative to FFM), or training volume (Table 1). Males and females did not differ with respect to Hb FSR (males: 0.80 [0.25]%/day; females: 0.88 [0.17]%/day; *p* = 0.43) and albumin FSR (males: 4.1 [1.4] %/day; females: 3.9 [0.3] %/day; *p* = 0.25). Hb ASR was also not different between sexes when examining absolute (males: 7.6 [2.6]; females: 5.7 [1.5] g/day; *p* = 0.11), BM‐normalized (males: 98 [29]; females: 97 [21] mg/kg BM/day; *p* = 0.93), or FFM‐normalized values (males: 124 [43]; females: 131 [31] mg/kg FFM/day; *p* = 0.67).

## 4 DISCUSSION

The aim of the present study was to apply the ^2^H_2_O method for measuring protein synthesis rates to investigate the influence of training status on Hb turnover in humans. Using the ^2^H_2_O method, the mean Hb synthesis rate was 0.84%/day translating to an estimated Hb lifespan of ~126 days; however, in contrast to our hypothesis, we observed no effect of training status on Hb FSR or Hb ASR. In our secondary analysis, we did not identify differences between sexes. Despite no effect of training status or sex on RBC production, the ^2^H_2_O method appears to be useful for quantifying Hb FSR and could facilitate future studies in human physiology related to erythropoiesis.

That we observed no difference in estimated RBC lifespan between trained and untrained individuals is contrary to previous findings. Weight et al. (1991) reported a mean RBC lifespan in trained runners (~70 days) that was considerably lower than untrained participants (~114 days). In contrast, the RBC lifespan we calculated from our Hb FSR measure was similar in trained and untrained individuals and comparable to their control group. One explanation could be that our trained group may not have been exercising as strenuously as that from Weight et al. (1991). While we recruited endurance‐trained individuals, they would not be classified as highly trained or elite athletes (McKay et al., 2022). Our two groups demonstrated divergent training habits and different fitness status, as suggested by both the training load data collected during the study period as well as V̇O_2_max and relative hematological values (Hb_mass_), which were significantly different between the two groups (Table 1), as expected (Heinicke et al., 2001; Kontro et al., 2024); however, when normalizing hematological variables to FFM, the differences between the groups were no longer significant, perhaps indicating that the size of the relative protein pool was not substantially different between groups in our sample. Compared with an untrained population, body mass‐normalized blood volume is expected to be ~10%, 20%, and 30% higher in moderately trained individuals, trained athletes, and elite athletes, respectively (Schmidt & Prommer, 2008), whereas the difference was only 17% in the present study. This discrepancy could also explain the similar Hb ASR, which would be expected to differ even with a similar Hb FSR if the protein pool size was greater in trained individuals. Alternatively, previous studies on runners instead of general endurance athletes could explain the discrepancy between studies: foot strike hemolysis, the destruction of RBCs from repeated striking of the ground while running (Telford et al., 2003) rather than aerobic training per se, may explain the much shorter RBC lifespan in the study from Weight et al. (1991). Whether the different methodological approaches explain the lack of differences between groups in our study is unclear but unlikely given the mean estimated RBC lifespan of our untrained group was similar to their control group.

While sex differences in RBC lifespan (or FSR) have not been reported to our knowledge, either sex could theoretically have a greater RBC lifespan. Firstly, increased RBC production may be necessary to offset blood lost through menstruation or withdrawal bleeding (in OC users) in reproductive age females, but females are also more likely than males to be iron deficient, which could limit the optimal rate of erythropoiesis (Finch & Cook, 1984). Male blood donors have higher estimated median densities for RBCs, indicating a higher biological cell age (Mykhailova et al., 2024), but male RBCs are more likely to hemolyze than female RBCs (Kanias et al., 2017), possibly due to the effect of estrogen on RBC rheology (Grau et al., 2018), potentially indicating a shorter lifespan. We found a numerically greater Hb FSR (and shorter RBC lifespan) in our female volunteers, but it is uncertain whether the nonsignificant finding is real or a type I error due to our limited sample size (powered for differences in training status rather than sex differences).

The derived average lifespan of 126 days is similar to the ~120‐day lifespan of RBCs that is often reported (Berlin et al., 1959; de Back et al., 2014; Shemin & Rittenberg, 1946; Zhang et al., 2018). Previous studies employed some form of cohort cell labeling, such as ^15^N ingestion, or the injection of cells labeled with radioactive di‐iso‐propyl‐fluorophosphonate (DP^32^F), ^51^Cr, or biotin (Bentley et al., 1974; Bratteby & Wadman, 1968; Khera et al., 2015; Mock et al., 2011). With previous studies, interindividual variation is considerable (i.e., SD > 20 days), which is congruent with the present data. Since we measured Hb FSR and not the destruction rate of released RBCs, our calculated lifespan is technically for Hb and not mature cells, adding ~7 days to the expected RBC lifespan assuming a constant rate of Hb synthesis during the 14‐day maturation. The primary advantages of the method we employed to assess Hb FSR compared to previous methods are that (i) the sampling protocol is much shorter (i.e., 1 vs. 6 months), reducing the duration of the study, participant burden, and opportunity for label recycling; (ii) the tracer is administered orally and is a stable isotope, as opposed to radioactive isotopes used in other protocols, making it safer and easier to use (Kontro et al., 2025). The assumptions for using this method in the manner we have employed—the validity of the precursor pool as a surrogate for bone marrow amino acid enrichment, the use of RBC protein to measure Hb FSR, and a lack of early RBC destruction—are reasonable. The ^2^H_2_O method will facilitate studies of erythropoiesis in response to experimental interventions and clinical conditions.

As intense exercise has been found to acutely stimulate albumin synthesis (Nagashima et al., 2000; Yang et al., 1998), it is plausible that albumin FSR would also be higher in athletes compared to non‐athletes. In contrast, we found a similar albumin FSR in our sample of trained and untrained individuals (3.95 vs. 4.08%∙day^−1^) determined from a 48‐h change in albumin APE, between days 1 and 3. Thus, although chronic endurance training does not seem to increase the turnover of albumin, our value aligned well with values previously reported in the literature (Fu & Nair, 1998; Gersovitz et al., 1980; Previs et al., 2004), providing some further reassurance that our Hb FSR data are valid.

In conclusion, the Hb FSR did not differ between endurance‐trained and untrained, healthy adults. In consideration of previous results from highly trained runners, these results could indicate that running, but not aerobic fitness per se, influences RBC lifespan in humans. Furthermore, the Hb FSR technique was feasible and yielded expected values, suggesting it could be useful for investigating erythropoiesis in humans in other contexts.

## AUTHOR CONTRIBUTIONS

All authors contributed to the conception and design of the work and the acquisition, analysis, and/or interpretation of the data. HK and MJM wrote the first draft of the manuscript, and all authors critically revised the manuscript for intellectual content. All authors approved the final version of the manuscript and agreed to be accountable for all aspects of the work in ensuring that questions related to the accuracy and integrity of any part of the work are appropriately investigated and resolved. All persons listed as authors qualify for authorship, and all those who qualify for authorship are listed.

## FUNDING INFORMATION

This work was supported by an operating grant from the Natural Sciences and Engineering Research Council of Canada (NSERC; grant number RGPIN‐2018‐06424), start‐up funding from the Faculty of Kinesiology (University of Calgary), and a University of Calgary VPR Catalyst Grant, all received by MJM. HK was supported by a Faculty of Kinesiology Dean's Doctoral Scholarship.

## CONFLICT OF INTEREST STATEMENT

The authors have no competing interests to declare.

## Data Availability

The data supporting the findings of this study are included within the figures or are available from the corresponding author upon reasonable request.
